# Time to suicide after psychiatric inpatient discharge: a nationwide Swedish survival analysis

**DOI:** 10.1186/s12888-026-07878-5

**Published:** 2026-02-05

**Authors:** Jonas Berge, Elin Fröding Saric, Tabita Sellin, Margda Waern, Åsa Westrin, Sara Lindström

**Affiliations:** 1 Department of Clinical Sciences, Lund, Psychiatry, Lund University, Lund, 22185 Sweden; 2 Addiction Centre Malmö, Malmö, 20502 Sweden; 3 Region Jönköpings län, Jönköpings University, Jönköping, Sweden; 4 Department of Psychiatry, University Health Care Research Centre, Faculty of Medicine and Health, Örebro University, Örebro, SE 70182 Sweden; 5 Psychosis Department, Sahlgrenska University Hospital, Region Västra Götaland, Göteborg, Mölndal SE-431 30 Sweden; 6 Department of Psychiatry, Skåne University Hospital, Lund, Sweden

**Keywords:** Suicide, Psychiatric discharge, Survival analysis, Risk factors

## Abstract

**Background:**

The period immediately following psychiatric inpatient care is recognized as a time of elevated risk of suicide, yet little is known about factors that influence how soon suicide occurs after discharge.

**Aim:**

To examine the timing of suicide among persons after psychiatric discharge and identify sociodemographic and clinical factors associated with a shorter time to death by suicide.

**Methods:**

We included all adults in Sweden who died by suicide in 2015 and who had been discharged from psychiatric inpatient care during the last three months of life (*n* = 140). Sociodemographic and clinical variables were extracted from electronic medical records, including contextual factors from the last hospitalization (involuntary care, documented suicide risk, unplanned discharge). Time to suicide was modelled using stratified Cox proportional hazards regression.

**Results:**

The median time from discharge to death was 32 days. Older age was the only background factor independently associated with a shorter time to suicide; each additional 10 years of age was associated with shorter time to suicide, as indicated by an increased unadjusted hazard ratio (uHR) of 1.26 (95% CI: 1.12–14.2, *p* < .001). A diagnosed neurotic, stress-related and somatoform disorder (ICD-10 code F40-F49) was associated with a longer time to suicide (uHR = 0.68 (0.47–0.98), *p* = .036), although this association was not statistically significant when adjusting for age (aHR 0.82, 0.56–1.20, *p* = .310). Previous suicide attempts, sex, substance use disorder and depressive disorders were not associated with time to suicide. Neither involuntary care nor the presence or absence of a formal suicide risk assessment during inpatient stay predicted earlier suicide. Unplanned discharges were likewise unrelated to the time to suicide.

**Conclusions:**

Among people who died by suicide within three months of psychiatric discharge, only older age independently predicted a shorter time to suicide. The absence of clear signals—particularly from routinely collected risk assessments—suggests that timely, universal follow-up may be more appropriate than attempts to target aftercare on the basis of standard risk markers.

**Clinical trial number:**

Not applicable.

**Supplementary Information:**

The online version contains supplementary material available at 10.1186/s12888-026-07878-5.

## Introduction

In a Swedish medical record review based on suicide deaths in 2015, 60% of people who died by suicide had contact with some type of healthcare provider in the four weeks preceding death, and 90.3% had contact within the previous two years [1], the settings included primary care, somatic (non-psychiatric) specialist care and psychiatric services, underscoring the central role of health professionals in identifying and responding to suicidal crises. Individuals who have recently received psychiatric inpatient care account for a disproportionately large share of suicides; a Swedish nationwide cohort study revealed that nearly half of all suicides occurred among people who had been hospitalised for psychiatric care during the two years prior to death [2] and in the nationwide cohort underlying the present study, approximately 70% of suicide decedents had been admitted to psychiatric inpatient care during the two years preceding death, while about 12% had been discharged within the final three months of life. Meta-analyses consistently report that suicide risk is extremely high immediately following psychiatric discharge and declines rapidly over time [3]. The meta-analysis calculated pooled suicide rates of approximately 2950 suicides per 100,000 person-years during the first week after discharge, decreasing to 2060 per 100,000 during the first month and further to approximately 494 per 100,000 over one to five years. Earlier reviews noted that suicide rates in the first three months after discharge are approximately 15 times higher than the population rate and that the risk remains elevated for several years [4]. Large registry-based cohort studies support this pattern. The largest Swedish cohort study to date, covering nearly 2.9 million discharges, identified 3695 suicides within 30 days after discharge [2]. The risk was especially high among patients with depression and those who had shown suicidal behaviour within 30 days prior to admission. Elevated suicide risk has also been reported following discharge from somatic care. A nationwide Norwegian study revealed that individuals discharged after acute physical health hospitalization had a seven-fold increase in suicide risk during the first four weeks [5].

Psychiatric admission practices have changed substantially over recent decades, with shorter inpatient stays, higher thresholds for admission, and an increased emphasis on outpatient and community-based care [6–8]. However, despite these secular changes, psychiatric discharge remains a well-established period of particularly elevated suicide risk, underscoring the continued relevance of examining temporal patterns to suicide following inpatient care.

Multiple studies have investigated risk factors for suicide after psychiatric discharge, including previous suicide attempts, suicidal ideation, male sex and depressive disorders. However, a recent meta-analysis concluded that these clinical factors have modest effects and do not explain why the risk is so strongly elevated in the first period following discharge [3]. Structured risk assessments are widely used in clinical practice, but their predictive validity remains uncertain. Evidence suggests that post discharge follow-up may mitigate risk; a U.S. cohort study of adolescents revealed that follow-up care within seven days of discharge was associated with a 56% reduction in subsequent risk for suicide [9], yet nearly half of the patients did not receive such contact.

Despite extensive research, critical knowledge gaps persist. The underlying reasons for the persistently high post-discharge suicide rates remain unclear. First, there is a limited understanding of the temporal course of suicide after discharge: most studies report cumulative rates over broad periods without examining how risk evolves from discharge until the event. Second, no study in Sweden has explored whether clinicians’ structured suicide risk assessments are associated with time to suicide.

## Aims

To examine the timing of suicide after psychiatric discharge and identify sociodemographic and clinical factors associated with a shorter time to death by suicide. By clarifying when suicide occurs and whether risk assessments differentiate patients with shorter versus longer times to suicide, this can contribute to a deeper understanding of factors associated with the occurrence of rapid suicide after discharge and provide information for future prevention strategies.

## Methods and materials

The present study was conducted as part of a broader nationwide research initiative entitled *Retrospective investigation of health care utilization among individuals who died by suicide in Sweden in 2015* [1]. Information on deaths by suicide was obtained from the Cause of Death Register maintained by the National Board of Health and Welfare [10], where suicide is coded as the underlying cause of death according to the ICD-10 categories X60–X84 [11]. The year 2015 was selected because the study is based on a nationwide, manual medical record review covering all health care contacts prior to death, which required extensive regional coordination and investigator training. Complete national coverage of the Cause of Death Register and medical records for this year allowed a comprehensive and internally consistent dataset, whereas extending the review across multiple years was not feasible within available resources.

### Medical record review protocol

All available medical records from both public and private healthcare services were reviewed using a structured protocol specifically developed for this study. The research group created the protocol in accordance with the recommendations of the Swedish Psychiatric Association [12] and includes items covering various aspects of health care utilization.

### Data collection and confidentiality procedures

Access to medical records was managed regionally. Regional health care representatives signed confidentiality agreements with the project leader, ensuring compliance with Swedish patient confidentiality legislation. These representatives identified eligible cases in their respective regions and appointed local investigators, who also signed confidentiality agreements before gaining access to the records. All the investigators participated in a standardized training session on how to use the review protocol, organized by members of the research group. The training was held on multiple occasions as new investigators joined the project, and a written manual was provided to guide the review process. Continuous supervision and updates were offered throughout the data collection. The investigators were mainly clinicians familiar with the regional electronic health record systems. To ensure objectivity, the investigators did not review cases involving patients they had previously met in their clinical work.

### Study population

In total, 1,179 individuals who died by suicide in Sweden in 2015 were identified through the Cause of Death Register. Of these, 824 (69.9%) had received psychiatric inpatient care during the two years preceding their death. For the present study, we included all individuals who had been discharged from psychiatric inpatient care within the three months before the suicide (*n* = 140). In the case of patients having several inpatient admissions, we considered only the last discharge.

### Psychiatric inpatient admissions

Data were collected from medical records for the last psychiatric inpatient admission within the last three months before the suicide for individuals who had been discharged from psychiatric inpatient care within the last three months before death by suicide. The main variable of interest was time (in days) from discharge until suicide. We also collected information about whether any part of the last inpatient admission care was given involuntarily under the Swedish Involuntary Care Act, whether the discharge was performed according to a specified plan, and information regarding the last recorded consultation with a doctor during the inpatient admission. Specifically, the information gathered included whether the medical doctor recorded thoughts about death, thoughts about suicide, the presence of plans to die by suicide, and whether or not the overall evaluation at the last noted psychiatric assessment in inpatient care was an increased risk of suicide. Information on suicidal ideation and suicide risk assessment was derived from routine clinical documentation in the medical records and not from standardized research instruments. Specifically, data were extracted from physicians’ notes regarding documented thoughts about death, thoughts about suicide, presence of suicidal plans, and the clinician’s overall assessment of suicide risk at the most recent psychiatric inpatient assessment prior to discharge.

No uniform or mandatory set of questions was used to assess these items. Instead, documentation reflected routine clinical practice, where physicians are expected to assess suicidality using structured clinical judgement when relevant, particularly in psychiatric care. However, the use of specific questions or assessment formats is not mandated, and documentation practices may therefore vary between clinicians and clinical settings.

### Background data

Background data extracted from any of the previously described data sources were used in the study. The specific information used was sex (refers to registered biological sex female/male. For readability, the terms women and men are used in the text to refer to female and male sex, respectively), age (modelled per 10-year increase to improve interpretability), previous recorded suicide attempts (any during the lifetime and number of attempts during the past 12 months), total number of inpatient admissions for the past three months and previously recorded psychiatric diagnoses. The psychiatric diagnoses, which were coded via the ICD-10 system, were collapsed into the following broad categories: substance use disorders (F10–F19), psychotic disorders (F20–F29), bipolar disorders (F31), depressive disorders (F32–F33), neurotic, stress-related and somatoform disorders (F40–F49)personality disorders (F60), autism (F84), and Attention-Deficit/Hyperactivity Disorder (ADHD) (F90).

### Missing data

Owing to the presence of missing data in this small sample, a strategy for handling missing data was deemed necessary. Depending on the design of the data extraction protocol, it is, in many cases, reasonable to assume that missing data reflect the absence of the specific item in question. However, it is rarely possible to determine whether missing data resulted from a lack of information on the specific issue or the absence of the issue at hand. Furthermore, it cannot be determined whether the missing data are missing at random. Based on these considerations, missing data were therefore handled differently depending on the specific variable, which was determined on a case-by-case basis for each variable.

Missing data on previous suicide attempts were coded as no previous suicide attempts, as no documentation of prior attempts was found in the medical records (*n* = 12). Diagnoses were noted only as a string of ICD-10 codes recorded for each patient, so there were no formally missing data. For the data on the last psychiatric inpatient stay, admissions with missing data for involuntary care (*n* = 2) were coded as voluntary hospital stays. Missing data for unplanned discharge (*n* = 2) were coded as planned discharge. For the data from the last consultation with the doctor, the rates of missing data were high for thoughts about death (*n* = 37), suicidal thoughts (*n* = 18), suicide plans (*n* = 19), and global evaluation of suicide risk (*n* = 30). In the main analysis, we coded missing data for variables stemming from the last inpatient stay as phenomena not present. However, because of the centrality of these variables for our main research question, we also performed sensitivity analyses in which we excluded cases with missing data for these variables.

### Statistical analysis

The main statistical analysis method used was Cox regression, with time from discharge until suicide used as the survival time and suicide used as the event variable. This constitutes a complete case analysis, as all members of the study cohort had died by suicide, and there was thus no censoring. Cox regression using complete case data is a legitimate method of analysis, albeit with important limitations regarding the interpretation of the results [13]. The results of the present analysis should thus be interpreted as analysing factors associated with earlier suicide in the subset of patients who die by suicide within three months of discharge from a psychiatric inpatient stay.

Some patients might have had several inpatient admissions in the last three months. For these patients, earlier discharges from inpatient admissions might formally be considered discharges that did not lead to suicide but rather readmission. To control for this, we stratified all Cox regression analyses for the total number of psychiatric inpatient admissions in the past three months. Variables from the inpatient admission and background variables were entered in the analyses as independent variables, first one by one in unadjusted models, and then all variables with an unadjusted p-value of < 0.10 were entered together in an adjusted model. The proportional hazards assumption was tested for each covariate via Schoenfeld residuals, complemented by visual inspection of residual plots. Sensitivity analyses were performed with the listwise exclusion of individuals with missing data for the variables reflecting the last consultation with a doctor prior to discharge. All the statistical analyses were performed via the survival package in R 4.4.2 [14].

## Results

### Sample characteristics

Of the 140 individuals in the cohort, 91 (65%) were women and 49 (35%) were men (Table 1). The mean age was 48.5 years (range 16–94), and the median time from discharge to suicide was 32 days for women and 31 days for men, with substantial variability across individuals. A history of suicide attempts was recorded for 84 individuals, while 43 had no such history and 13 had missing data; this corresponds to 66.1% of cases when missing data are excluded (60.0% when included), and almost half had a documented suicide attempt within the last 12 months.

**Table 1:** Univariable cox regression analyses of time to suicide after discharge from inpatient psychiatric care (*n* = 140)

Variables		*n*	Time to suicide	uHR (95% CI)	*p* value
			(median, IQR)		
Sex					
	Female	91	32 (14–58)	1	
	Male	49	31 (9–64)	0.93 (0.65–1.33)	0.680
Age (per 10 years of age*)		140	-	1.27 (1.12–1.43)	< 0.001
Previous suicide attempts					
	Never	54	41 (14–64)	1	
	Yes, but not in the past 12 months	37	23 (12–54)	1.14 (0.74–1.75)	0.564
	In the past 12 months	49	35 (14–60)	0.93 (0.62–1.38)	0.713
Substance abuse disorders					
	No	101	35 (13–63)	1	
	Yes	39	23 (13–55)	0.92 (0.62–1.37)	0.694
Depressive disorder					
	No	119	33 (13–60)	1	
	Yes	21	23 (12–62)	1.00 (0.62–1.61)	0.997
Neurotic, stress-related and somatoform disorders					
	No	89	28 (10–54)	1	
	Yes	51	44 (19–69)	0.67 (0.47–0.97)	0.032
Psychotic disorders					
	No	130	32 (13–62)	1	
	Yes	10	33 (15–42)	0.97 (0.50–1.89)	0.935
Bipolar disorder					
	No	127	33 (13–62)	1	
	Yes	13	29 (14–54)	1.28 (0.71–2.29)	0.406
Personality disorder					
	No	128	32 (13–60)	1	
	Yes	12	53 (16–74)	0.73 (0.39–1.37)	0.327
Autism					
	No	136	32 (13–62)	1	
	Yes	4	28 (13–48)	1.00 (0.27–3.67)	0.996
ADHD					
	No	128	32 (13–61)	1	
	Yes	12	41 (19–66)	0.69 (0.36–1.30)	0.249

Note. uHR = univariable hazard ratio; CI = confidence interval. *age divided by 10 to facilitate interpretation

*Univariable analyses*

According to the univariable Cox regression models (Table 2), older age was significantly associated with a shorter time to suicide. In contrast, having a neurotic, stress-related or somatoform disorder was associated with a longer time to suicide. Other factors, including sex, previous suicide attempts, substance use disorders, depressive disorders, and other diagnostic categories, were not significantly related to the timing of suicide. Neither involuntary care nor being discharged without a planned follow-up was associated with time to suicide. Moreover, documentation of suicidal thoughts, suicide plans, or elevated suicide risk at the last doctor consultation during the final admission was not associated with time to suicide.

**Table 2:** Univariable cox regression analyses of variables recorded during the last doctor consultation prior to discharge

Variables		*n*	Time to suicide	uHR (95% CI)	*p* value
			(median, IQR)		
Involuntary care					
	No	103	31 (13–55)	1	
	Yes	37	48 (10–69)	0.83 (0.56–1.23)	0.36
Unplanned discharge					
	No	110	30.5 (13-59.25)	1	
	Yes	30	42 (13.25–70.5)	1.01 (0.66–1.53)	0.98
Thoughts about death					
	No	114	32.5 (13.25–63.75)	1	
	Yes	26	23 (9-51.75)	1.21 (0.77–1.9)	0.408
Suicide thoughts					
	No	117	32 (13–62)	1	
	Yes	23	35 (9-58.5)	1.14 (0.7–1.84)	0.605
Suicide plans					
	No	129	32 (13–63)	1	
	Yes	11	35 (14.5–48)	1.2 (0.62–2.35)	0.585
Elevated suicide risk					
	No	105	33 (15–62)	1	
	Yes	35	22 (6.5–52)	1.22 (0.81–1.82)	0.341

Note. uHR = univariable hazard ratio; CI = confidence interval

*Multivariable analysis*

**Fig. 1:**
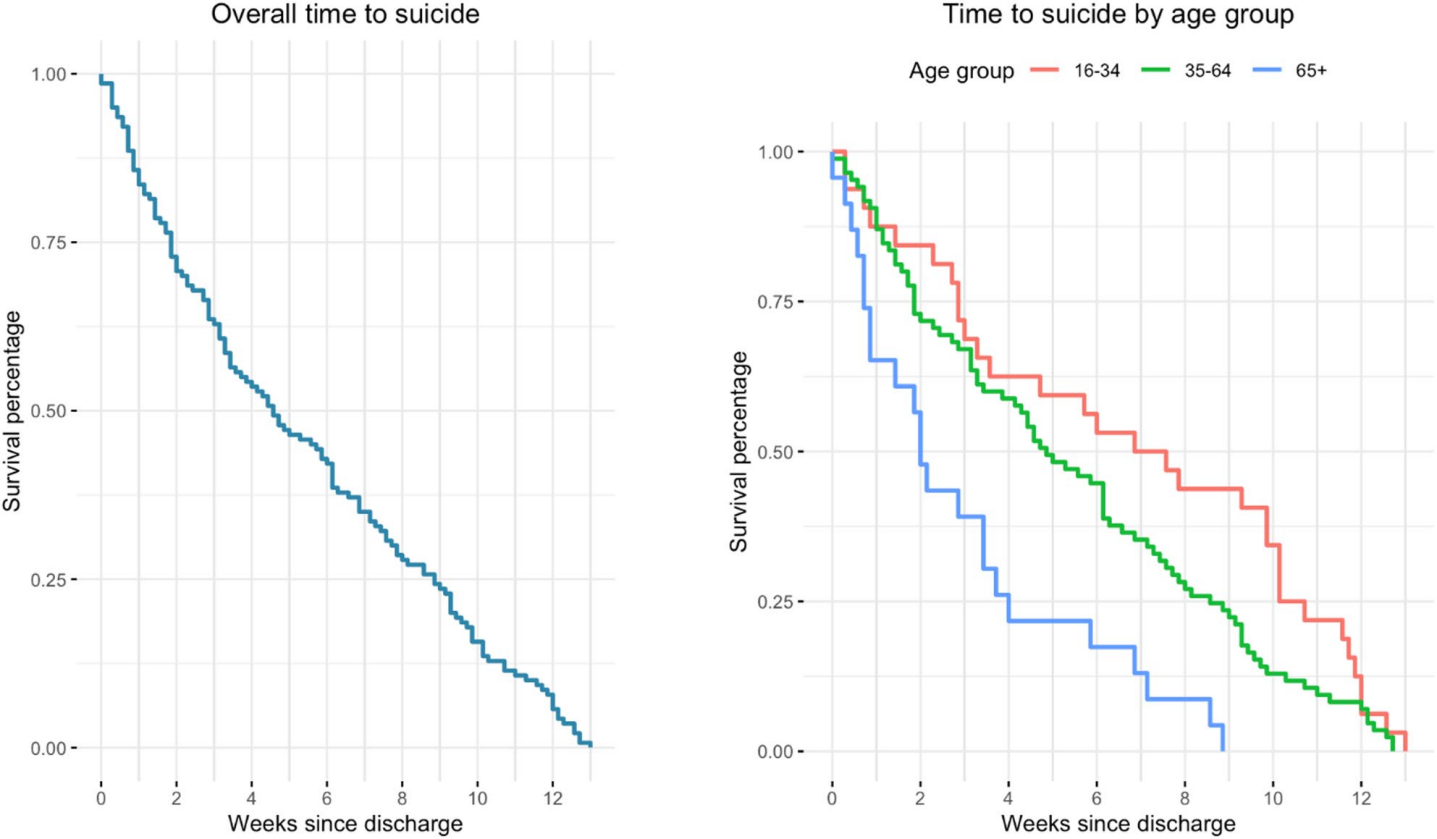
Time to suicide following discharge from psychiatric inpatient care

When the significant variables in univariable analysis age and neurotic, stress-related and somatoform disorders were included in a multivariable model (Table 3), only older age remained independently associated with a shorter time to suicide. This association is illustrated in Fig. 1. As shown by the age-stratified survival curves, individuals aged 65 years and older exhibited a more rapid decline in survival following discharge, indicating a shorter time to suicide compared with individuals aged 35–64 years and 16–34 years. The association between neurotic, stress-related and somatoform disorders and longer times to suicide observed in the univariable analysis was attenuated and no longer statistically significant in the multivariable model.

**Table 3:** Multivariate Cox regression analyses

Variables		*n*	aHR (95% CI)	*p* value
Age (per 10 years of age)		140	1.24 (1.09–1.4)	0.001
Neurotic, stress-related and somatoform disorders				
	No	89	1	
	Yes	51	0.82 (0.56–1.2)	0.310

Note. aHR = adjusted hazard ratio; CI = confidence interval

The left panel shows the overall survival curve for time to suicide among all individuals included in the study. The right panel displays survival curves stratified by age group (16–34 years, 35–64 years, and ≥ 65 years). A steeper decline in the survival curve indicates a shorter time to suicide. Individuals aged 65 years and older show a consistently shorter time to suicide following discharge compared with younger age groups.

### Sensitivity analysis

In the sensitivity analysis for the analyses presented in Table 2, we performed the same analyses but with listwise exclusion of individuals with missing data for the variables reflecting the last consultation with a doctor prior to discharge. The results did not differ substantially from the main results (Table S1, supplementary material).

## Discussion

This study sought to clarify when suicide occurs after discharge from psychiatric inpatient care and whether routinely collected risk markers predict a shorter time to suicide. The present analyses were designed to examine temporal discrimination within an ultra–high-risk group, rather than to inform individual-level clinical decision-making at discharge. In our cohort of patients who died by suicide within three months of discharge, the median time to death was 32 days, indicating that fatalities often occur within a relatively short window after individuals leave the hospital. According to the univariable analyses, older age was associated with a shorter time to suicide, and a neurotic, stress-related or somatoform disorder predicted a longer time. After adjusting for age, the association with neurotic, stress-related and somatoform disorders was not statistically significant. Notably, previous suicide attempts, sex, substance use disorder, depressive disorder, involuntary admission, formal suicide risk assessments and unplanned discharge were not associated with time to suicide.

To contextualise these findings beyond the Swedish setting, they should be interpreted in relation to international evidence on age, sex, and diagnostic patterns in suicide following psychiatric and emergency care discharge. Large register-based studies consistently demonstrate that suicide risk is markedly elevated during psychiatric inpatient stay and in the immediate post-discharge period, with particularly high absolute rates observed among men and older adults. In a Danish nationwide study, Madsen et al. [15] reported extreme suicide rates during admission and in the first week following discharge, with the highest rates among individuals diagnosed with affective disorders and anxiety- and stress-related disorders, while comparatively lower rates were observed among those with schizophrenia spectrum disorders. Although men exhibited higher absolute suicide rates, women showed higher relative risks compared with women without prior admission, illustrating that sex differences vary depending on the risk metric applied.

Similar patterns have been reported in other European settings. In a 15-year cohort study of psychiatric discharges, König et al. [16] found that affective disorders and neurotic stress-related disorders were associated with substantially increased post-discharge suicide risk, whereas schizophrenia spectrum and personality disorders were not significantly associated with suicide in adjusted analyses. Male sex and older age were independently associated with increased suicide risk, but diagnostic, age, and sex effects were modelled as time-independent predictors of suicide occurrence and were not examined in relation to temporal proximity to suicide.

Population-based evidence from Spain further supports these observations. Mortier et al. [17] reported markedly elevated suicide risk following discharge from psychiatric inpatient care, particularly during the first year, with increased risk associated with depressive disorders, adjustment disorders among men, and bipolar disorder among women. Older age and male sex were consistently associated with higher suicide risk, yet, as in previous studies, these factors were examined in relation to overall suicide occurrence rather than timing of suicide during follow-up. Evidence from emergency care settings also indicates that suicide may occur very shortly after discharge. In a retrospective study of adults discharged from a hospital emergency department, Maestre-Orozco et al. [18] found that suicide within 30 days of discharge, although rare in absolute terms, often occurred within the first few days, predominantly among men, and frequently in individuals presenting with somatic complaints. Psychiatric diagnoses, most commonly depression or anxiety, were common but not universal, underscoring that suicidality may remain unrecognised at the time of discharge.

Taken together, these studies demonstrate that age, sex, and diagnostic category are robust markers of *overall* suicide risk following discharge across health care systems, with higher risk consistently observed among men, older individuals, and those diagnosed with affective or anxiety-related disorders. However, across this literature, these factors are primarily used to identify populations at elevated risk and are not examined in relation to temporal variation in suicide occurrence among individuals who ultimately die. In this context, our findings extend existing knowledge by showing that, within a population consisting exclusively of suicide decedents, most diagnostic categories and sex were not associated with shorter time to suicide, while older age was associated with a more rapid progression to suicide after discharge. This suggests that age, sex, and diagnosis are important indicators of vulnerability at the population level but have limited discriminatory value for predicting temporal proximity to suicide within an already ultra–high-risk group, highlighting the limitations of risk stratification approaches based solely on static patient characteristics.

An important characteristic of this cohort is the predominance of women (65%), which contrasts with national suicide statistics in which men account for more than two-thirds of deaths by suicide. This difference is likely related to help-seeking patterns and service utilization. Thus, the present findings should be interpreted in light of a selected subgroup of suicide decedents, rather than the broader population of individuals who die by suicide. Another notable feature of this cohort is the high proportion of individuals with previous suicide attempts. This is expected, given that a history of suicide attempts is one of the strongest predictors of future suicidal behavior [19], and such individuals are more likely to be admitted for psychiatric inpatient care prior to death.

The findings are consistent with prior evidence that suicide is concentrated in the early post discharge period [3], but they add nuance by showing that the timing of death among those who die is not strongly predicted by the most commonly cited risk factors. A recent meta-analysis [3] reported that commonly assessed clinical factors, such as prior self-harm, suicidal ideation, and depressive symptoms, have only modest predictive value for *overall suicide occurrence* after discharge when comparing patients who die by suicide with those who do not. While these findings pertain to risk prediction at the population level, they underscore the broader limitations of clinical risk factors for distinguishing suicide outcomes, which is conceptually consistent with our observation that such factors were not informative for differentiating *timing* to suicide among individuals who ultimately died.

Older age was associated with a shorter time to suicide following discharge. Rather than reflecting a fixed difference in time to suicide, this age effect appears to indicate a temporal clustering of suicides shortly after discharge among older adults. The association between older age and a shorter time to suicide may involve several mechanisms. Older individuals may have more severe somatic illness trajectories, greater hopelessness, or service-related issues, including limited availability to geropsychiatric specialists or lower engagement in post discharge care. In addition to clinical and health care–related factors, social isolation may represent an important underlying mechanism linking older age to a shorter time to suicide. Older adults are more likely to live alone and to experience the loss of a partner, friends, or family members, which may reduce access to emotional support and opportunities for external intervention during periods of crisis. Previous research [20, 21] has shown that social isolation, loneliness, and bereavement are strongly associated with suicide risk in later life, and may contribute to more rapid progression from suicidal thoughts to suicidal behaviour by limiting protective social factors. Studies in general population samples have shown that suicide risk increases with age, particularly among men [22]. In contrast, the tentative association between neurotic, stress-related and somatoform disorders and a longer time to suicide might indicate that such patients experience protracted periods of ambivalence or recurrent crises, delaying the act. However, this association did not withstand adjustment for age, and the small sample size limits definitive conclusions. A formal assessment of elevated suicide risk at the last physician contact prior to discharge was not associated with time to suicide. One possible explanation is that when clinicians identify acute and imminent risk, they respond by delaying discharge or intensifying safety measures, including safety planning, increased observation, or adjustments in treatment. Such actions may reduce the likelihood of suicide in the immediate post-discharge period, even if longer-term vulnerability remains. This finding illustrates a central tension in contemporary suicide prevention: on the one hand, statutory guidelines call for structured risk assessments based on group-level risk factors, resulting in categorical classifications such as low, moderate, or high risk. On the other hand, empirical evidence demonstrates that these categories have limited sensitivity and specificity [23–27], and fail to capture the dynamic and fluctuating nature of suicidality. Suicidal thoughts and impulses can shift rapidly in response to changes in mood, interpersonal stressors or life events, and static risk scores can therefore neither reflect this variability nor provide sufficient guidance for clinical decision-making. Taken together, these findings highlight the need to move beyond prediction-oriented risk stratification and toward therapeutic, patient-centred approaches that emphasize continuous assessment, engagement and personalized safety measures.

### Strengths, limitations and future directions

Important strengths of this study include the use of the Cause of Death Register combined with detailed chart reviews, which allowed precise measurement of time to death and examination of a range of clinical factors. However, several limitations merit consideration. The sample included only individuals who died by suicide within the first three months after discharge, and associations with time to death may differ in the wider cohort of all discharged patients. The modest sample size (*n* = 140) limits the statistical power for less common diagnoses and restricted multivariable analyses. Variables such as suicidal intent, hopelessness or social support—factors that may change rapidly—were not available. Finally, the data were drawn from 2015, and the use of data from a single calendar year may limit generalisability if patterns of psychiatric care or discharge practices have changed over time. However, there is no indication that the fundamental features of post-discharge suicide risk—namely the concentration of deaths in the early post-discharge period—have altered substantially in recent decade. The focus of the present study on temporal proximity to suicide rather than on service utilisation rates or treatment effects further reduces sensitivity to incremental changes in clinical practice over time.

Future research should replicate these findings in larger samples and across longer follow-up periods, including individuals who survive after discharge, to identify dynamic predictors of timing. Implementation science is needed to test various forms of follow-up and safety planning interventions in routine psychiatric practice. Additionally, research should investigate how to integrate real-time monitoring (e.g., ecological momentary assessment) into post discharge care to identify emerging suicidal crises and tailor interventions.

## Conclusions

Among Swedish patients who die by suicide within three months of psychiatric discharge, older age was the only clinical predictor of shorter time to death. Frequent recent healthcare contact, along with major deficiencies in risk assessment, underscores the limitations of existing risk assessments and highlights the need for proactive, collaborative, and universal post discharge interventions. Refining the focus from prediction to early engagement and therapeutic management may offer a more effective route toward reducing suicide mortality in this high-risk period.

## Supplementary Information

Below is the link to the electronic supplementary material.

Supplementary Material 1

## Data Availability

The datasets generated and analysed during the current study are not publicly available owing to confidentiality and Swedish ethical regulations, but are available from the corresponding author upon reasonable request.
