# Supplementary material for: Time to suicide after psychiatric inpatient discharge: a nationwide Swedish survival analysis
Source: BMC Psychiatry. 2026 Feb 5;26:213. doi: 10.1186/s12888-026-07878-5 (PMC12930994; doi:10.1186/s12888-026-07878-5)
Supplement: Supplementary file 1 — Supplementary Material 1 [file 12888_2026_7878_MOESM1_ESM.docx]

Supplementary material

The table below mirrors table 2 in the paper, but with cases with missing data excluded from analysis.

| **Table S1.** Univariable Cox regression analyses of variables recorded during the last doctor consultation prior to discharge as in table 2, but with listwise exclusion of cases with missing data. | | | | | |
| --- | --- | --- | --- | --- | --- |
| Variables | | n | Time to suicide | uHR (95% CI) | p value |
| Total | | 140 |  |  |  |
| Involuntary care | |  |  |  |  |
|  | No | 103 | 31 (13-55) | 1 |  |
|  | Yes | 37 | 48 (10-69) | 0.83 (0.56-1.23) | 0.350 |
| Unplanned discharge | |  |  |  |  |
|  | No | 108 | 30.5 (13-57.75) | 1 |  |
|  | Yes | 30 | 42 (13.25-70.5) | 0.93 (0.62-1.41) | 0.744 |
| Thoughts about death | |  |  |  |  |
|  | No | 77 | 28 (13-57) | 1 |  |
|  | Yes | 26 | 23 (9-51.75) | 1.2 (0.76-1.91) | 0.437 |
| Suicide thoughts | |  |  |  |  |
|  | No | 99 | 31 (13.5-56.5) | 1 |  |
|  | Yes | 23 | 35 (9-58.5) | 1.16 (0.72-1.88) | 0.537 |
| Suicide plans | |  |  |  |  |
|  | No | 110 | 31 (12.25-59.25) | 1 |  |
|  | Yes | 11 | 35 (14.5-48) | 1.16 (0.59-2.27) | 0.662 |
| Elevated suicide risk | |  |  |  |  |
|  | No | 75 | 30 (14-58.5) | 1 |  |
|  | Yes | 35 | 22 (6.5-52) | 1.16 (0.76-1.77) | 0.492 |
